# A precise machine learning model: Detecting cervical cancer using feature selection and explainable AI

**DOI:** 10.1016/j.jpi.2024.100398

**Published:** 2024-09-26

**Authors:** Rashiduzzaman Shakil, Sadia Islam, Bonna Akter

**Affiliations:** Department of Computer Science and Engineering, Daffodil International University, Dhaka, Birulia 1216, Bangladesh

**Keywords:** Cervical cancer, SMOTE, ADASYN, Chi-square, LASSO, Machine learning, Decision tree, Explainable AI, SHAP

## Abstract

Cervical cancer is a cancer that remains a significant global health challenge all over the world. Due to improper screening in the early stages, and healthcare disparities, a large number of women are suffering from this disease, and the mortality rate increases day by day. Hence, in these studies, we presented a precise approach utilizing six different machine learning models (decision tree, logistic regression, naïve bayes, random forest, k nearest neighbors, support vector machine), which can predict the early stage of cervical cancer by analysing 36 risk factor attributes of 858 individuals. In addition, two data balancing techniques—Synthetic Minority Oversampling Technique and Adaptive Synthetic Sampling—were used to mitigate the data imbalance issues. Furthermore, Chi-square and Least Absolute Shrinkage and Selection Operator are two distinct feature selection processes that have been applied to evaluate the feature rank, which are mostly correlated to identify the particular disease, and also integrate an explainable artificial intelligence technique, namely Shapley Additive Explanations, for clarifying the model outcome. The applied machine learning model outcome is evaluated by performance evaluation matrices, namely accuracy, sensitivity, specificity, precision, f1-score, false-positive rate and false-negative rate, and area under the Receiver operating characteristic curve score. The decision tree outperformed in Chi-square feature selection with outstanding accuracy with 97.60%, 98.73% sensitivity, 80% specificity, and 98.73% precision, respectively. During the data imbalance, DT performed 97% accuracy, 99.35% sensitivity, 69.23% specificity, and 97.45% precision. This research is focused on developing diagnostic frameworks with automated tools to improve the detection and management of cervical cancer, as well as on helping healthcare professionals deliver more efficient and personalized care to their patients.

## Introduction

Cervical cancer arises primarily in the cervix cells located in the lower region of the uterus. Infection with human papillomaviruses (HPVs), transmitted through sexual contact, is the primary cause of cervical cancer. According to data, cervical cancer ranks first or second among women for cancer-related deaths.^1^ A significant proportion of the recorded cases in 2018 originate from both impoverished and industrialized nations.^2^ Cervical cancer constitutes 6.5% of all malignancies in women. Around 342,000 cervical cancer deaths and 604,127 new cases of the illness are expected to occur globally in 2020.^3^ The prevalence of cervical cancer is a significant public health issue, especially in affluent nations: The number of newly identified instances of invasive cervical cancer in Europe is 54,517 each year, with 24,874 women losing their lives to this type of cancer.^4^

Vaccinations are commonly included in standard immunization regimens. Approximately, 90% of European girls are expected to have had the full HPV vaccination by the age of 15 by 2030.^5^ Moreover, the World Health Organization (WHO) strongly emphasizes the need of European decision-makers intensifying their efforts to eradicate cervical cancer by utilizing existing preventive measures.^6^ Systematic screenings and prompt detection, like other medical conditions, can significantly reduce the likelihood of mortality.^7^ Given the lack of symptoms during the first phases of the disease, the identification of cervical cancer may provide challenges. Systematic annual examinations often uncover alterations in the cervix. The most common sign of cervical cancer is abnormal bleeding, which can change in severity as the disease progresses. In approximately 90% of cases, the advanced phases of the illness exhibit distinct hallmarks.^8^ Common signs of this condition include reddish discharge, spotting, contact bleeding, and postmenopausal bleeding. In addition to the symptoms, the existence of bloody discharge, which usually has an unpleasant smell, is another crucial indication of cervical cancer. When the disease has evolved to a certain point, it can affect neighboring organs, which can lead to lower abdominal discomfort.^9^

To reduce vast mortality rate, early detection of the disease is essential. Early-stage cervical cancer can be cured at an average rate of 80% with either radical surgery or radiation.^10^

One of the most well-known branches of artificial intelligence (AI), machine learning, has been quite successful in many different areas.^11^ Machine learning algorithms are now being used more and more to predict cervical cancer and its early stages by utilizing several forms of data, such as cytology (pap smear), histology, scanning, and clinical information. AI has demonstrated significant potential in accurately predicting cervical cancer by examining several datasets linked to the condition. The development of explainable AI (XAI) originated from the critical need for ensuring responsible deployment of machine learning models. It acknowledges the issue that intricate algorithms can frequently function as ‘black boxes’, rendering their decision- making procedures obscure and potentially resulting in biases or ethical concerns.

This study focuses on the utilization of XAI approaches, specifically SHAP, to enhance the comprehension of cervical cancer predictions. Recognition of this information is essential for healthcare professionals to accurately evaluate the model's results and optimize the prediction process for better accuracy and clinical significance. This study utilized machine learning models to predict the probability of negative and positive cervical cancer for clinical use.

The contribution of this study are as follows:

• Introducing two robust feature selection strategies, including the Chi-square test and Least Absolute Shrinkage and Selection Operator (LASSO), to improve the predictive accuracy and efficiency of cervical cancer prediction models.
• In this research, a comparative result analysis was presented between two distinct data balancing techniques, namely synthetic minority oversampling technique (SMOTE) and Adaptive Synthetic Sampling (ADASYN).
• In addition, we conducted a novel study where we integrated the explainable AI model, SHAP, to improve the interpretability and transparency of our cervical cancer prediction models.
• This study utilized six machine learning models, such as decision trees (DTs), logistic regression (LR), naive bayes (NB), random forest (RF), k nearest neighbor (KNN), and support vector machines (SVMs). Each of these models contains distinct advantages and qualities, rendering them ideal for certain elements of the prediction task.
• The model's performance metrics were measured in terms of accuracy, specificity, sensitivity, F1-score, precision, false-positive rate (FPR), and false-negative rate (FNR) to thoroughly evaluate their ability to make accurate predictions.

## Background and literature review

Nowadays, cervical cancer is one of the most alarming diseases for women in society. Therefore, researchers are presenting their own methodology utilizing AI to identify the early stages of cervical cancer.

Mehmood et al.^12^ proposed a novel approach named CervDetect for detecting cervical cancer using machine learning algorithms. They used pearson correlation to identify relevant features. The RF and shallow neural networks were utilized to accurately predict cervical cancer and achieved an accuracy of 93.6% and mean square error of 0.07111. Arora et al.^13^ introduced a method for classifying Herlev pap smear image datasets using SVMs. In their study, bilateral filtering was used for noise removal and local Gaussian fitting energy segmentation for cell and nuclei segmentation, achieving 95% accuracy.

Sabanci et al.^14^ classify patients based on their survey responses to identify the high risk of cervical cancer using multilayer perceptron, BayesNet, and KNN methods. They achieved the highest result of false positives in the BayesNet and KNN algorithms. A study on the risk factors associated with cervical cancer conducted by Razali et al.^15^ They utilized classification algorithms namely NB, DT, and KNN. This study applied mean to handling the missing values and SMOTE for data balancing. They evaluated the effectiveness of several classifiers using 10-fold cross-validation, showcasing the potential of data mining techniques in medical diagnosis and treatment planning.

Parikh et al.^16^ developed an approach to predicting cervical cancer utilizing machine learning algorithms and various factors present in the dataset. Their study includes the establishment of three models: KNN, DT, and RF. Specially, the KNN achieved the highest accuracy among the three models. This research showed the capability to identify individuals at risk of cervical cancer. Ou et al.^17^ employed six machine learning classifiers to predict postoperative pathological risk factors in 1260 early-stage cervical cancer patients, who had a radical hysterectomy. The RF classifier demonstrated the highest level of accuracy in predicting deep stromal infiltration, achieving an accuracy rate of 70.8% and an AUC (area under the receiver operating characteristic (ROC) curve) value of 0.767.

Malli et al.^18^ proposed an automated machine learning approach using KNN and ANN to predict cervical cancer. The technique analyzes the color and shape features of the cervix cell nucleus and cytoplasmic segments using a fuzzy-based technique. They achieved 88.04% accuracy with kNN and 54% with ANN for cell classification in cervical cancer prediction. Latha et al.^19^ compared the data mining algorithms for classifying and identifying the stage of cervical cancer. These algorithms were analyzed by accuracy, sensitivity, and specificity, and J48 was found to be the most effective with 93.03% specificity and sensitivity over 80%. A DT accurately identifies attributes closely related to cancer staging, with sensitivity surpassing 70%.

Sun et al.^20^ proposed a technique for identifying cervical cancer by employing a RF classifier along with ReliefF feature selection. The study utilizes preprocessing, segmenting, and extracting 20 features and ranks these features by importance. The RF classifier, utilizing the most significant top 13 features, outperformed NB, C4.5, and LR, achieving 94.44% accuracy and AUC score of 98.04%. Ilyas et al.^21^ presented a novel ensemble classification approach to enhance the diagnostic precision of cervical cancer. By employing an ensemble method that combines the predictions of various classifiers including DT, SVM, RF, K-NN, NB, MLP, J48, and LR. This study highlights the capacity of machine intelligence to provide cost-effective and rapid diagnosis of cervical cancer.

Nithya et al.^22^ presented an efficient framework for cervical cancer diagnosis by addressing challenges related to high-dimensional, redundant, and imbalanced datasets. Their goal is to achieve classification accuracy and computational efficiency by combining filter- and wrapper-based feature selection methods. Vidya et al.^23^ analyzed three supervised machine learning methods: ID3, C4.5, and NB, for cervical cancer prediction using a novel NCBI dataset. ID3 and C4.5 build DTs iteratively, whereas NB quickly creates Bayesian statistical models. Overall, NB predicts cervical cancer better than other classifiers.

Tseng et al.^24^ applied machine learning techniques to predict cervical cancer recurrence-proneness to improve clinical decision-making. They used medical records and pathology data to identify recurrence risk factors, finding that the C5.0 model is particularly effective. This study suggested pathological stage and pathological T for better surveillance and treatment based on these predictive labels. Chaudhuri et al.^25^ suggested a three-stage hybrid feature selection approach and a stacked classification model for early detection of cervical cancer using machine learning techniques. In this research, six algorithms have been employed including LR, NB, SVM, ET, RF, and GDB, where NB outperformed with 97%.

Yang et al.^26^ explored machine learning models to improve the diagnosis and prediction of cervical cancer using MLP and RF. They identified age, number of sexual partners, and hormonal contraception as major risk factors for cervical cancer. To conduct this study, they used 858 individuals information. Kalbhor et al.^27^ developed a method for diagnosing cervical cancer by classifying cytology pap smear images. This study analyzed the efficacy of utilizing discrete cosine transform and Haar transform coefficients as characteristics for image classification. They employed seven ML classifiers where the RF classifier achieved the highest accuracy rate of 81.11%. Table 1. consists outcome of the related research work which are studied by several researcher.

**Table 1:** Comparative analysis with other existing work.

Author and reference	Dataset source	Data frequency	Applied feature selection	Best ML classifier	Performance outcome
Mehmood et al.^12^	UCI Machine Learning Repository	859	Random forest, Pearson correlation	RF and shallow neural network	ACC: 93.6%,
Arora et al.^13^	Herlev University Hospital. Denmark	917	Principal component analysis (PCA)	SVM	ACC: 95%
Sabanci et al.^14^	UCI Machine Learning Repository	858	No	NB	ACC: 97%
Razali et al.^15^	UCI Machine Learning Repository	858	No	RF	ACC: 96.40%
Parikh et al.^16^	UCI Machine Learning Repository	429	Hill climbing algorithm	KNN	ACC: 95.3%.
Ou et al.^17^	Wenzhou Medical University	1260	LinearSVC with L1 penalty	RF	ACC: 70.8%
Malli et al.^18^	UCI Machine Learning Repository	300	PCA	KNN	ACC: 88.04%
Latha et al.^19^	Indian Statistical Institute, Calcutta	203	CFS	J48	ACC: 93.03%
Sun et al.^20^	Herlev University Hospital, Denmark	917	ReliefF	RF	ACC: 94.44%
Ilyas et al.^21^	UCI Machine Learning Repository	858	Cross-validation and supervised feature selection	Ensemble classifier	ACC: 94%
Nithya et al.^22^	UCI Machine Learning Repository	Not mentioned	Information gain, Chi-square Correlation coefficient, RFE, GA, Sequential feature selection	RF	ACC: 85.5%
Vidya et al.^23^	National Center for Biotechnology Information (NCBI)	Not mentioned	PCA	NB	ACC: 81%
Tseng et al.^24^	Chung Shan Medical University Hospital Tumor Registry.	168	GainRatio	C5.0	ACC: 96%
Chaudhuri et al.^25^	UCI Machine Learning Repository	858	Multistage GA	GDB	ACC: 97%
Yang et al.^26^	Caracas University Hospital in Venezuela	858	GA	MLP	ACC: 97.2%
Kalbhor et al.^27^	Herlev University Hospital, Denmark	917	DCT, Haar Transform	RF	ACC: 81.11%
**Shakil et al. (proposed methodology)**	**UCI Machine Learning Repository**	**858**	**Chi-square and LASSO**	**DT**	**ACC: 97.60%**

## Methodology

**Fig. 1:**
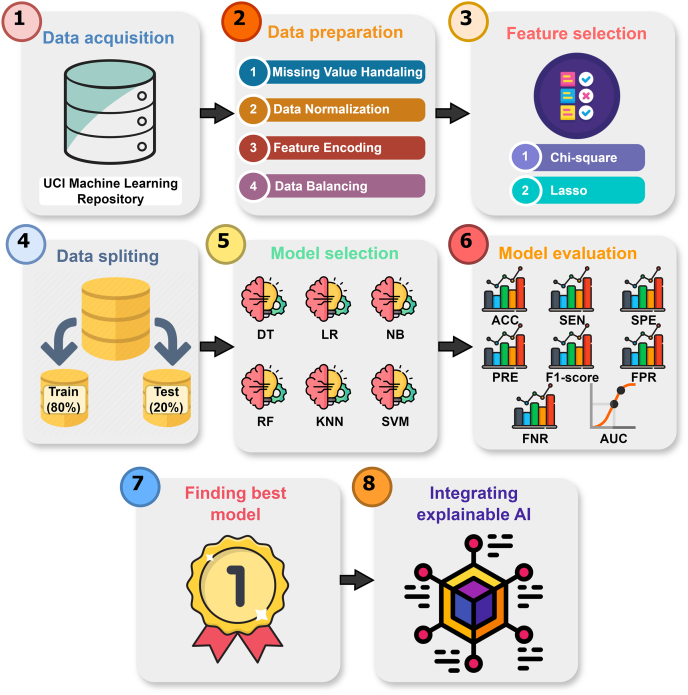
Visualization the overall working procedure of this study.

In this section, we are mentioned all required task sequentially. Fig. 1 illustrates the overall working flow that is divided into seven sections: data acquisition, data preparation, feature selection, data split, model selection, evaluation of the applied model, finding the best model, and integrating explainable AI.

### Data collection

**Fig. 2:**
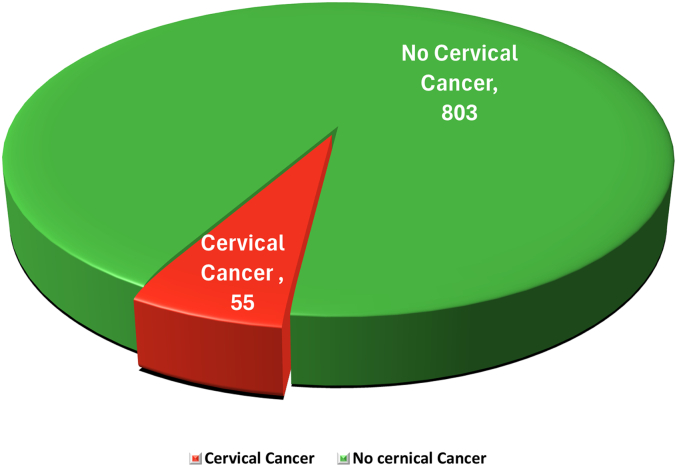
Ratio of cervical cancer and no cervical cancer based on overall dataset.

To conduct this research, we used cervical cancer (risk factors) dataset, which was collected from a renowned online repository called UCI machine learning repository.^28^^,^^29^ The dataset was collected at ‘Hospital Universitario de Caracas’ in Caracas, Venezuela. This dataset comprises 858 individual records of medical patients, each with 36 distinct attributes, of which 55 belong to the cervical cancer class and 803 do not have cervical cancer. Fig. 2. depicts the ration of dataset between two classes. The primary feature of this dataset is its ability to distinguish cervical cancer patients based on their demographic information, habits, and historical medical records.

### Data preprocessing

**Fig. 3:**
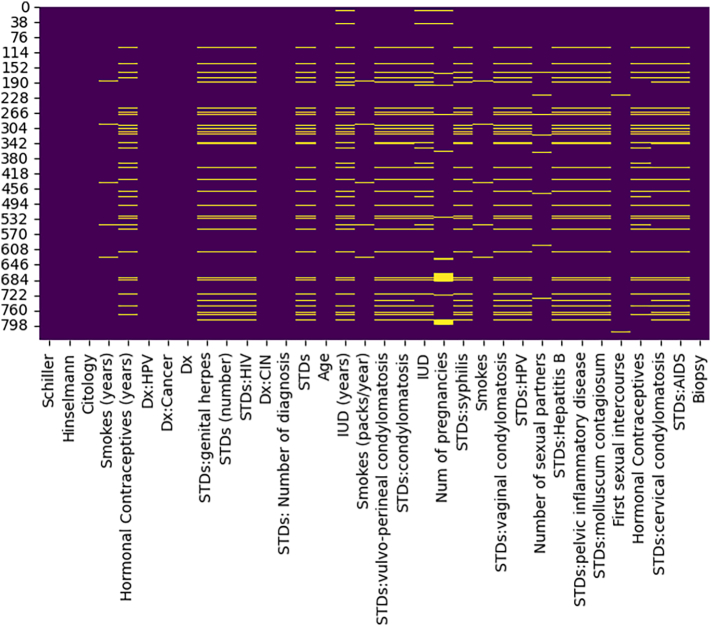
Number of missing values of each attributes.

Data preprocessing is a crucial part of machine learning; it can improve data quality, enhance the performance of models, and ensure data consistency. During this phase, at first, we checked the missing values by applying the isnull() function and handling the missing values using mean.^30^ Fig. 3 visualizes the number of missing values of each features. Secondly, a min-max scaler was used to normalize this dataset, and the range of the normalization was from 0 to 1. Thirdly, we employed feature encoding to convert our target class (biopsy class) into 0 and 1, where cervical cancer is 1 and not 0. Furthermore, the SMOTE and ADASYN are two different data balancing techniques that had been used to eliminate the class imbalance problem. SMOTE aims to balance class distribution by generating synthetic examples for the minority class.^31^ ADASYN also balances class distribution, but it focuses on generating synthetic data points that are harder to classify, thus improving the learning of complex decision boundaries.^32^ Moreover, the whole dataset was separated into training and testing with a ratio of 80:20.

### Feature selection

Feature selection is the process of identifying the most significant features from a large number of attributes that are not relevant for this particular disease. In this study, we employed two different feature selection techniques, named LASSO and Chi-square. LASSO feature selection utilizes regression technique to identify and select the most relevant features for classifying cervical cancer presence or absence.^33^ Table 2 contain the selected features by Chi-square and LASSO. Initially, this dataset consists of 36 features. During feature selection phase, Chi-square feature selection has 30 features and 17 features was selected by LASSO feature selection. This approach operates by imposing a penalty on the coefficients of the regression model, hence causing certain coefficients to be reduced to zero. In addition, it can improve the performance of the models and interpretability by removing less-essential elements. Within the framework of binary classification, the LASSO technique assists in differentiating between malignant and benign instances by prioritizing the most influential variables. This ultimately contributes to a more precise and efficient diagnosis of cervical cancer.

(1) $$\underset{\beta }{\mathrm{minimize}}\left(\frac{1}{n}\sum_{i=1}^{n}\left[y_{i}\log\left(h_{\beta }\left(X_{i}\right)\right)+\left(1-y_{i}\right)\log\left(1-h_{\beta }\left(X_{i}\right)\right)\right]+\lambda \sum_{j=1}^{p}|\beta_{j}|\right)$$

**Table 2:** Features rank according to the feature section technique.

Method used	Features
Chi-square	Schiller, Hinselmann, Citology, Smokes (years), Hormonal contraceptives (years), Dx:HPV, Dx:Cancer, Dx, STDs:genital herpes, STDs (number), STDs:HIV, Dx:CIN, STDs: Number of diagnosis, STDs, Age, IUD (years), Smokes(packs/year), STDs: Vulvo-perineal condylomatosis, STDs:condylomatosis, IUD, Num of pregnancies, STDs: Syphilis, Smokes, STDs:vaginal condylomatosis, STDs:HPV, Number of sexual partners, STDs: Hepatitis B, STDs:pelvic inflammatory disease, STDs:molluscum contagiosum, First sexual intercourse
LASSO	Schiller, Hinselmann, Citology, Dx:HPV, Dx:Cancer, STDs:genital herpes, Dx:CIN, Hormonal Contraceptives (years), Number of sexual partners, Num of pregnancies, First sexual intercourse, STDs:vulvo-perineal condylomatosis, Smokes(packs/year), STDs: Vaginal condylomatosis, Number of diagnosis, IUD, STDs:Hepatitis B

Here, *n* is the total number of samples in dataset, *p* is the feature. Let *X* be the *n*×*p* feature matrix, *y* be the binary target vector, and β be the vector of coefficients.

where:

• *y*_*i*_ is the main target value for the *i*-th sample.
• β_0_ is the intercept.
• *X*_*ij*_ is the value of the *j*-th feature for the *i*-th sample.
• β_*j*_ is the coefficient for the *j*-th feature.
• λ is the regularization parameter that controls the amount of shrinkage applied to the coefficients.

The degree of independence between each characteristic and the target variable is assessed by computing the Chi-square statistic.^34^ A higher degree of dependence on the target variable indicates that the features are more significant and so are chosen.

For Chi-square feature selection in a binary classification task, where both feature *X* and the target *Y are binary*, the Chi-square statistic (χ^2^) measured how much obeserved distribution of the feature values differs from the expected distribution. The Chi-square statistic is calculated by the following formula:

(2) $$\chi^{2}=\sum_{i=1}^{r}\sum_{j=1}^{c}\frac{{\left(O_{\mathrm{ij}}-E_{\mathrm{ij}}\right)}^{2}}{E_{\mathrm{ij}}}$$

where:

The number of rows (categories of feature *X*) indicated by *r*. *c* is the number of columns (binary classes of target *Y*). *O*_*ij*_ is the observed frequency count of the *i*-th category of *X* and *j*-th class of *Y*. *E*_*ij*_ is the expected frequency count, calculated as:

(3) $$E_{\mathrm{ij}}=\frac{\sum_{k=1}^{r}O_{\mathrm{kj}}\sum_{l=1}^{c}O_{\mathrm{il}}}{N}$$

where *N* is the total number of observations.

### Applied machine learning model

In order to detect the presence of cervical cancer, this research employed six machine learning algorithms including DT, LR, NB, RF, KNN, and SVM. The details of applied model are mentioned below:

**Decision tree:** DT is a supervised learning model that is used for both classification and regression tasks. For binary classification of cervical cancer, the model splits the data into subsets based on the values of input features, recursively creating branches until reaching the leaf nodes, which represent the output class labels. The splitting is done using criteria like Gini impurity or entropy (information gain).^35^ Gini impurity is a measure of how often a randomly chosen element would be incorrectly labeled if it was randomly labeled according to the distribution of labels in the subset. It is calculated as:

(4) $$\text{Gini}=1-\sum_{i=1}^{C}p_{i}^{2}$$

where *p*_*i*_ is the probability of a randomly chosen element being classified as class *i*, and *C* is the number of classes (in binary classification, *C*=2).

Entropy used to evaluate best feature and threshold for splitting.

(5) $$\text{Entropy}=-\sum_{i=1}^{C}p_{i}\log_{2}\left(p_{i}\right)$$

where *p*_*i*_ is the probability of a randomly chosen element being classified as class *i*, and *C* is the number of classes.

**Logistic regression (LR):** One statistical model that is commonly employed for binary classification is LR. The likelihood that an input *x* falls into the positive category (cervical cancer) is represented by it. After applying the logistic (sigmoid) function to a linear combination of the input features, the output is a probability value between 0 and 1.^36^

(6) $$P\left(Y=1|X\right)=\frac{1}{1+e^{-\left(\beta_{0}+\beta_{1}x_{1}+\beta_{2}x_{2}+\cdots +\beta_{n}x_{n}\right)}}$$

where β_0_ is the intercept, β_*i*_ are the coefficients for each feature *x*_*i*_, and $X=\left(x_{1},x_{2},\ldots ,x_{n}\right)$ is the feature vector.

**Naïve bayes (NB):** The NB classifier is a probabilistic classification method that operates under the ‘naive’ premise that all features are independent of the class label. It determines the posterior probability of every class and forecasts the class with the highest probability for binary classification of cervical cancer.^37^

(7) $$P\left(Y|X\right)\propto P\left(Y\right)\prod_{i=1}^{n}P\left(x_{i}|Y\right)$$

where *P*(*Y*) is the prior probability of the class, and *P*(*x*_*i*_|*Y*) is the likelihood of feature *x*_*i*_ given class *Y*.

**Random forest:** RF is an ensemble learning technique that builds numerous DTs during training and produces the most frequent prediction among them for classification tasks.^38^ It enhances precision and mitigates overfitting by averaging many complex DT trained on different subsets of the same dataset.

(8) $$\hat{y}=\text{mode}\left(\left\{h_{1}\left(x\right),h_{2}\left(x\right),\ldots ,h_{B}\left(x\right)\right\}\right)$$

where *h*_*i*_(*x*) is the prediction of the *i*-th tree for input *x*.

**K-nearest neighbor (KNN):** KNN is a non-parametric, instance-based learning algorithm that classifies a sample based on the majority class among its *k* nearest neighbors in the feature space. For binary classification of cervical cancer, it considers the closest *k* samples in the training data.^39^

(9) $$\hat{y}=\text{mode}\left(\left\{y_{i}:x_{i}\in NN_{k}\left(x\right)\right\}\right)$$

where *N*_*k*_(*x*) represents the set of *k* nearest neighbors of *x*, and *y*_*i*_ is the class label of neighbor *x*_*i*_.

**Support vector machine (SVM):** SVM is a separating hyperplane-defined discriminative classifier. SVM uses support vectors that are closest to a hyperplane in high-dimensional space to increase the margin between cervical cancer binary classes.^40^

(10) $$f\left(x\right)=\mathrm{sign}\left(w\cdot x+b\right)$$

where **w** is the weight vector, **x** is the feature vector, and *b* is the bias term. The function sign determines the class label based on the sign of the result.

### Explainable artificial intelligence (XAI)

XAI is of significant importance in the healthcare field, because it is necessary to comprehend the reasoning behind the decisions made by machine learning models in order to facilitate their acceptance and use in clinical settings. Shapley Additive Explanations (SHAP), a type of XAI approach, improves the clarity and comprehensibility of AI models.^41^ These techniques assist in understanding the influence of factors like age, smoking habits, and HPV status on the model's decision to classify a sample as high- or low-risk in the context of cervical cancer classification. Model behavior can be better understood with the use of XAI, which in turn improves patient outcomes, enhances integrity in behavior, and secures compliance with regulations. Therefore, the incorporation of XAI in healthcare not only enhances the precision and dependability of AI applications but also encourages their adoption and utilization in clinical environments.

The SHAP value can be evaluated by following formula:

(11) $$\phi_{i}=\phi_{0}+\sum_{j=1}^{p}\omega_{j}z_{\mathrm{ij}}$$

where:

• $\phi_{i}$ represents the SHAP value for the *i*-th feature.
• $\phi_{0}$ is the base value, which is the average prediction of the model.
• $\omega_{j}$ are weights that represent the contribution of each feature.
• *z*_*ij*_ are binary variables (0 or 1) that indicate whether the *i*-th feature for the *j*-th instance is active.

### Performance evaluation metrices

Performance evaluation metrics are the integral part of assessing the effectiveness and efficiency of applied models. It offers a clear understanding of a model's performance in comparison to other models, enabling the selection of the most suitable model for a given task. In this research, we assess seven performance evaluation metrics to determine the model efficiency, and all of the metric formulas are mentioned below:

**Accuracy:** Accuracy is a metric that evaluates the overall correctness of a model by determining the proportion of correctly predicted cases, including both true positives (TPs) and true negatives (TNs), out of the total instances.

(12) $$\text{Accuracy}=\frac{\mathrm{TP}+\mathrm{TN}}{\mathrm{TP}+\mathrm{TN}+\mathrm{FP}+\mathrm{FN}}$$

**Sensitivity:** Sensitivity quantifies the accuracy of the model in correctly identifying actual positive cases. It is alternatively referred to as recall or the genuine positive rate.

(13) $$\text{Sensitivity}=\frac{\mathrm{TP}}{\mathrm{TP}+\mathrm{FN}}$$

**Specificity:** Specificity is a metric that quantifies the accuracy of a model in correctly identifying negative cases.

(14) $$\text{Specificity}=\frac{\mathrm{TN}}{\mathrm{TN}+\mathrm{FP}}$$

**Precision:** Precision is defined as the fraction of positive forecasts that are correct.

(15) $$\text{Precision}=\frac{\mathrm{TP}}{\mathrm{TP}+\mathrm{FP}}$$

**F1-score:** The F1-score represents the harmonic mean of precision and recall. It gives a single statistic that balances precision and recall, which is particularly beneficial when both are required.

(16) $$F1-\text{score}=2\times \frac{\text{Precision}\times \text{Recall}}{\text{Precision}+\text{Recall}}$$

**False-positive rate (FPR):** The FPR quantifies the ratio of negative cases that are erroneously identified as positive.

(17) $$\mathrm{FPR}=\frac{\mathrm{FP}}{\mathrm{FP}+\mathrm{TN}}$$

**False-negative rate (FNR):** The FNR quantifies the percentage of TP instances that are inaccurately identified as negative.

(18) $$\mathrm{FNR}=\frac{\mathrm{FN}}{\mathrm{FN}+\mathrm{TP}}$$

The proper meaning of TP, TN, FP, and FN are mentioned sequentially in below:

• TP: TP occurs when a model predicts cervical cancer and the actual condition is also cervical cancer.
• TN: TN would happen if a model predicts no cervical cancer and the actual condition is also no cervical cancer.
• FP: This occurs when a model predicts cervical cancer, but the actual condition is no cervical cancer.
• False negative (FN): The FN would happen if a model predicts no cervical cancer but the actual condition is cervical cancer.

## Result

In this section, we present the performance outcomes of six machine learning algorithms—DT, LR, NB, RF, KNN, and SVM—applied to the binary classification of cervical cancer. We evaluate each model based on key metrics, including accuracy, sensitivity, specificity, precision, F1-score, FPR, FNR, and AUC. Additionally, SHAP is an explainable AI technique that have been utilized for model interpretability, offering insights into the significance of various features. These results aim to highlight the comparative effectiveness of each algorithm in accurately distinguishing between healthy and cancerous cases, thereby informing the potential application of these models in clinical settings.

Table 3 represents the performance results of applied models without data balancing. It highlights the strengths and weaknesses across different metrics. The DT and RF algorithms both achieved high accuracy (97%), with DT excelling in sensitivity (99.35%) and RF showing a strong balance between sensitivity (97.50%) and specificity (85.71%). The LR performed accuracy rates (94.01%) with 96.82% precision. The NB algorithm achieved 92.21% accuracy, which is the lowest among all applied machine learning algorithms to classify cervical cancer patients. However, KNN and SVM both showed accuracy of 95.81%, with SVM having a slightly higher specificity (80%) compared to KNN (71.43%).

**Table 3:** Performance evaluation result without balancing.

Algorithm name	Accuracy (%)	Sensitivity (%)	Specificity (%)	Precision (%)	F1-score (%)	FPR (%)	FNR (%)
DT	97	99.35	69.23	97.45	98.39	30.77	0.65
LR	94.01	96.82	50	96.82	96.82	50	3.18
NB	92.21	96.75	38.46	94.90	95.82	61.54	3.25
RF	97	97.50	85.71	99.36	98.42	14.29	2.50
KNN	95.81	96.88	71.43	98.73	97.79	28.57	3.12
SVM	95.81	96.29	80	99.36	97.80	20	3.71

The summarizes performance evaluation results after data balancing with SMOTE is presented in Table 4. The LR algorithm achieved an accuracy of 94.61%, precision of 94.90%, and an F1-score of 97.06%. NB showed an accuracy of 86.22%, sensitivity of 98.55%, specificity of 27.59%, and an F1-score of 92.20%. KNN demonstrated a sensitivity of 99.34%, specificity of 56.25%, and precision of 95.54%. SVM exhibited the highest accuracy at 96.41%, sensitivity of 99.35%, specificity of 64.29%, precision of 96.82%, and an F1-score of 98.06%. These results highlight the effectiveness of SMOTE in improving classification metrics across different algorithms, with SVM achieving the highest overall accuracy.

**Table 4:** Performance evaluation result after data balancing using SMOTE.

Algorithm name	Accuracy (%)	Sensitivity (%)	Specificity (%)	Precision (%)	F1-score (%)	FPR (%)	FNR (%)
DT	86.23	98.57	29.62	87.97	92.96	70.38	1.43
LR	94.61	99.33	52.94	94.90	97.06	47.06	0.67
NB	86.22	98.55	27.59	86.62	92.20	72.41	1.45
RF	95.21	98.69	57.14	96.18	97.42	42.86	1.31
KNN	95.21	99.34	56.25	95.54	97.41	43.75	0.66
SVM	96.41	99.35	64.29	96.82	98.06	35.71	0.65

Table 5 presents the performance evaluation results after data balancing using ADASYN. The DT algorithm achieved 87.43% accuracy, 98.75% sensitivity, 29.62% specificity, and a precision of 87.97%. LR showed an accuracy of 89.22%, 93.99% of F1-score and 89.81% precision, and an FPR is 66.67%. RF gained a sensitivity rate of 99.34%, specificity of 56.25%, precision of 95.54%, and the FPR is 43.75%. The SVM algorithm achieved the highest accuracy at 96.41%, with a sensitivity of 99.35%, 64.29% specificity, 96.82% precision, and F1-score is 98.06%. Among the algorithms, SVM had the highest accuracy, whereas NB had the lowest accuracy of 85.63%.

**Table 5:** Performance evaluation result after data balancing using ADASYN.

Algorithm name	Accuracy (%)	Sensitivity (%)	Specificity (%)	Precision (%)	F1-score (%)	FPR (%)	FNR (%)
DT	87.43	98.57	29.62	87.97	92.96	70.38	1.43
LR	89.22	98.60	33.33	89.81	93.99	66.67	1.4
NB	85.63	98.54	26.67	85.99	91.84	73.33	1.46
RF	95.21	99.34	56.25	95.54	97.41	43.75	0.66
KNN	91.62	99.31	40.91	91.72	95.36	59.09	0.69
SVM	96.41	99.35	64.29	96.82	98.06	35.71	0.65

The study assessed the performance of the binary classification model by utilizing a confusion matrix, which is an essential tool for summarizing the predicted accuracy of the model. The confusion matrix is a tabular representation of a 2 × 2 table that presents the TP, TN, FP, and FN.

**Fig. 4:**
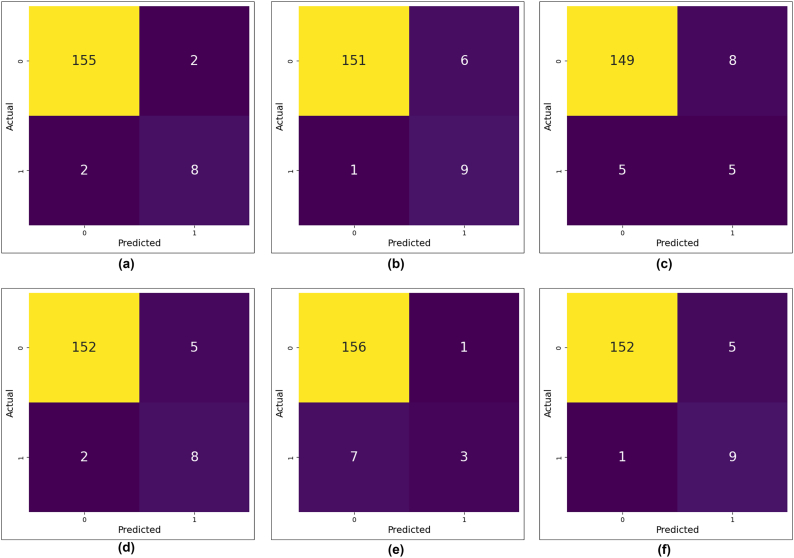
Confusion matrix of six machine learning algorithm in Chi-square feature selection, (a) DT (b) LR (c) NB (d) RF (e) KNN (f) SVM.

**Fig. 5:**
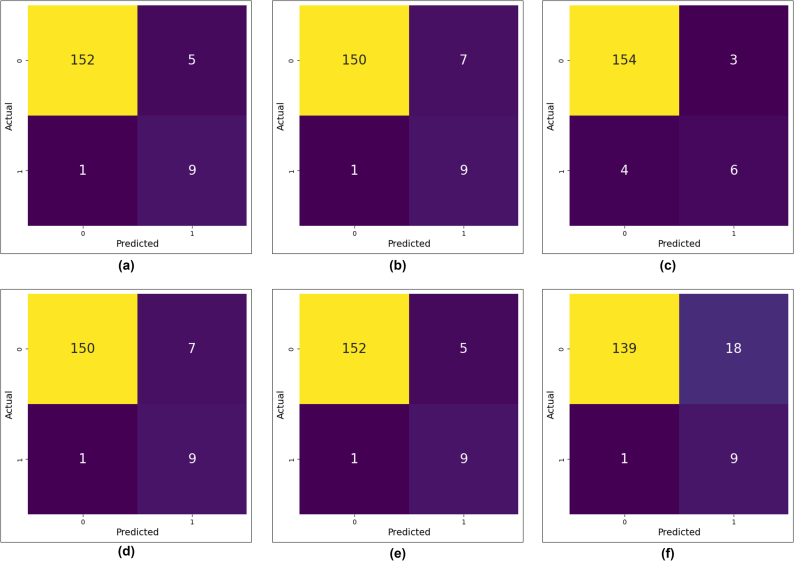
Confusion matrix of six machine learning algorithm in LASSO feature selection, (a) DT (b) LR (c) NB (d) RF (e) KNN (f) SVM.

Fig. 4, Fig. 5 depict the confusion matrix for Chi-square and LASSO feature selection for the six ml algorithms. In Fig. 4, DT has TP (155), TN (8), FP (2), and FN (2). The SVM classifier gained TN (9), TP (152), FP (5), and FN (1). In addition, in Fig. 5, DT and KNN have the same TP, TN, FP, and FN values of 152, 9, 5, and 1. Similarly, the performance of the LR algorithm is comparable to that of the RF.

Table 6 presents the performance evaluation results utilizing Chi-square feature selection. The DT algorithm achieved an accuracy of 97.60%, with a sensitivity of 98.73%, a specificity of 80%, and a precision of 98.73%. LR demonstrated an accuracy of 95.81%, an F1-score of 97.73%, a precision of 96.18%, an FPR of 40%, and an FNR of 0.66%. RF showed 98.70% sensitivity, 61.54% specificity, 96.82% precision, and 36.46% of FPR. NB performed with 92.21% accuracy, whereas it has 94.90% precision, 95.81% F1-score, 61.54% FPR, and 3.25% FNR. SVM had an accuracy of 96.41% with a sensitivity of 99.35%, and the specificity, precision, and F1-score were gradually 64.29%, 96.82%, and 98.06%, respectively. Among the algorithms, DT had the highest accuracy, making it the best performer. SVM, with an accuracy of 96.41%, ranked second. NB and KNN had the lowest accuracy at 92.21%.

**Table 6:** Performance evaluation result utilizing Chi-square feature selection.

Algorithm name	Accuracy (%)	Sensitivity (%)	Specificity (%)	Precision (%)	F1-score (%)	FPR (%)	FNR (%)
DT	97.60	98.73	80	98.73	98.73	20	1.27
LR	95.81	99.34	60	96.18	97.73	40	0.66
NB	92.21	96.75	38.46	94.90	95.81	61.54	3.25
RF	95.81	98.70	61.54	96.82	97.75	36.46	1.3
KNN	95.21	95.71	75	99.36	97.50	25	4.29
SVM	96.41	99.35	64.29	96.82	98.06	35.71	0.65

The performance evaluation results utilizing LASSO feature selection is present in Table 7. The DT and KNN algorithm have the similar performance result. The accuracy of 96.41%, where the sensitivity, specificity, precision, and F1-score are 99.35%, 64.29%, 96.82%, and 98.06%, respectively. The LR and RF algorithm gained the similar accuracy rate is 95.21%, and other performance evaluation results: sensitivity (99.34%), specificity (56.25%), FPR (43.75%), FNR (0.66%), and precision (95.54%). SVM indicate the lowest performance to classify the cervical cancer with an accuracy of 88.62%, 99.29% sensitivity, 33.33% specificity, and 91.90% F1-score. Among the algorithms, both DT and KNN had the highest accuracy at 96.41%, making them the best performers. NB with an accuracy of 95.81%, ranked in the second. LR and RF had the lowest accuracy at 95.21%.

**Table 7:** Performance evaluation result utilizing LASSO feature selection.

Algorithm name	Accuracy (%)	Sensitivity (%)	Specificity (%)	Precision (%)	F1-score (%)	FPR (%)	FNR (%)
DT	96.41	99.35	64.29	96.82	98.06	35.71	0.65
LR	95.21	99.34	56.25	95.54	97.40	43.75	0.66
NB	95.81	97.47	66.67	98.08	97.78	33.33	2.53
RF	95.21	99.34	56.25	95.54	97.40	43.75	0.66
KNN	96.41	99.35	64.29	96.82	98.06	35.71	0.65
SVM	88.62	99.29	33.33	85.54	91.90	66.67	0.71

**Fig. 6:**
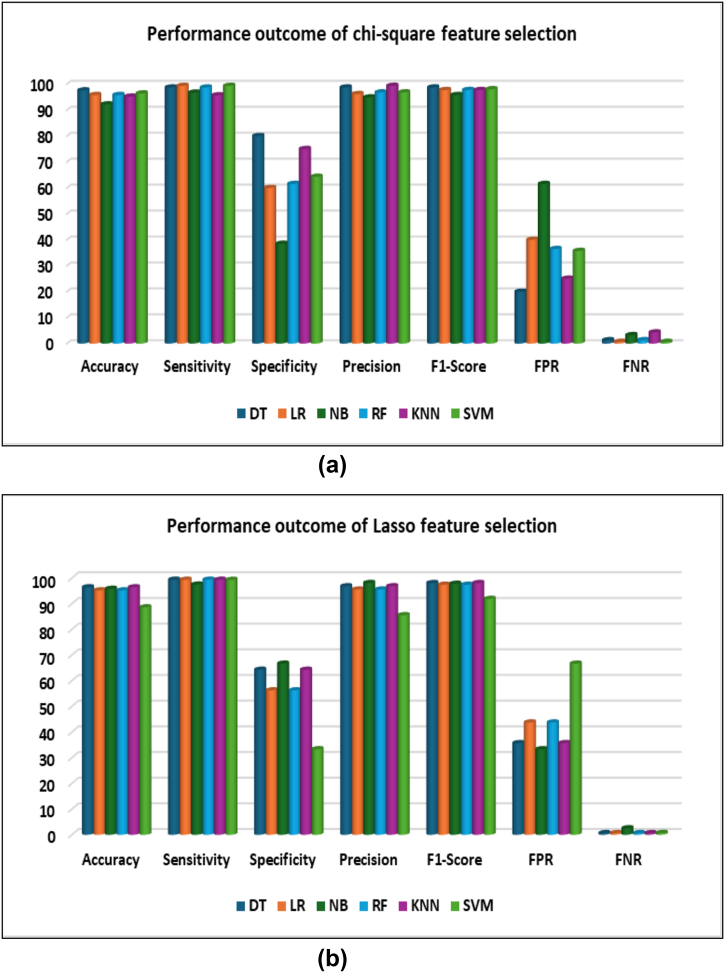
Comparative result visualization (a) Chi-square feature selection (b) LASSO feature selection.

Fig. 6 demonstrates overall performance outcome of Chi-square and LASSO feature selection. In this figure, the performance evaluation results -accuracy, sensitivity, specificity, precision, F1-score, FPR, and FNR are visualized in a bar graph. It is clear that DT algorithm gained 97.60% accuracy in Chi-square, which outreached other applied ml model performance.

In this study, we analyzed the performance of binary classification of cervical cancer using the ROC curve—a plot that illustrates the trade-off between the TPR and the FPR at various threshold settings—on the *x* and *y* axes. The TPR measures the proportion of actual positive cases (cervical cancer) correctly identified by the model, whereas the FPR represents the proportion of actual negative cases incorrectly classified as positive.

**Fig. 7:**
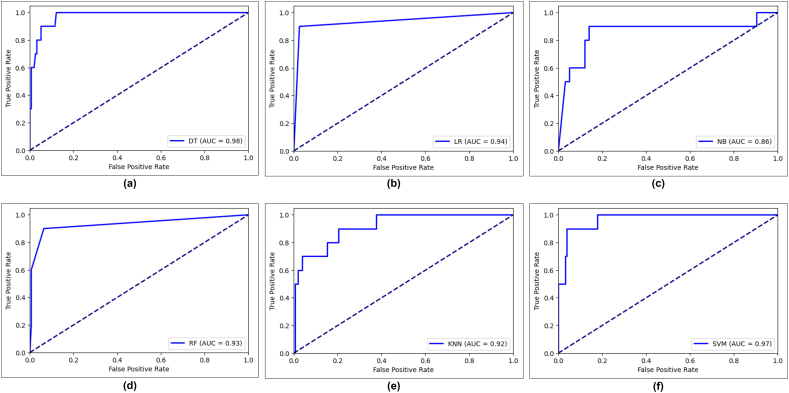
Receiver operating characteristic (ROC) curve of six machine learning algorithm in Chi-square feature selection, (a) DT (b) LR (c) NB (d) RF (e) KNN (f) SVM.

**Fig. 8:**
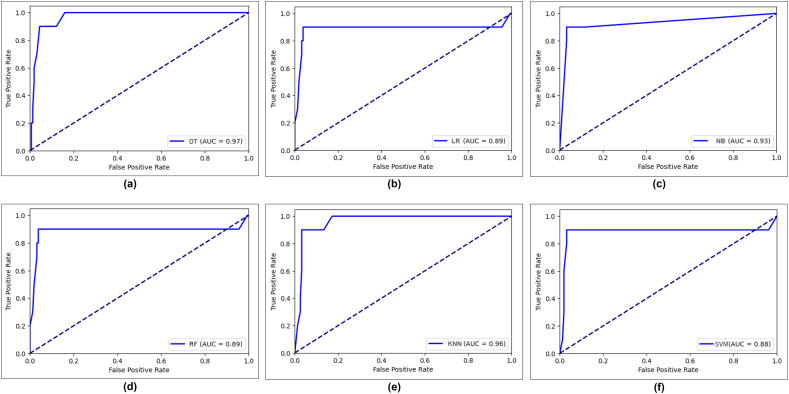
Receiver operating characteristic (ROC) curve of six machine learning algorithm in LASSO feature selection, (a) DT (b) LR (c) NB (d) RF (e) KNN (f) SVM.

Fig. 7, Fig. 8 depict the ROC score for Chi-square and LASSO feature selection for applied machine learning models. For Chi-square feature selection, the LR, NB, RF, KNN, and SVM are performed gradually at 0.94, 0.86, 0.93, 0.92, and 0.97, whereas DT outperformed with a 0.98 AUC score. Furthermore, after performing LASSO feature selection, the AUC scores of LR and RF have the same result of 0.89. By contrast, the DT algorithm gained 0.97, which is superior.

**Fig. 9:**
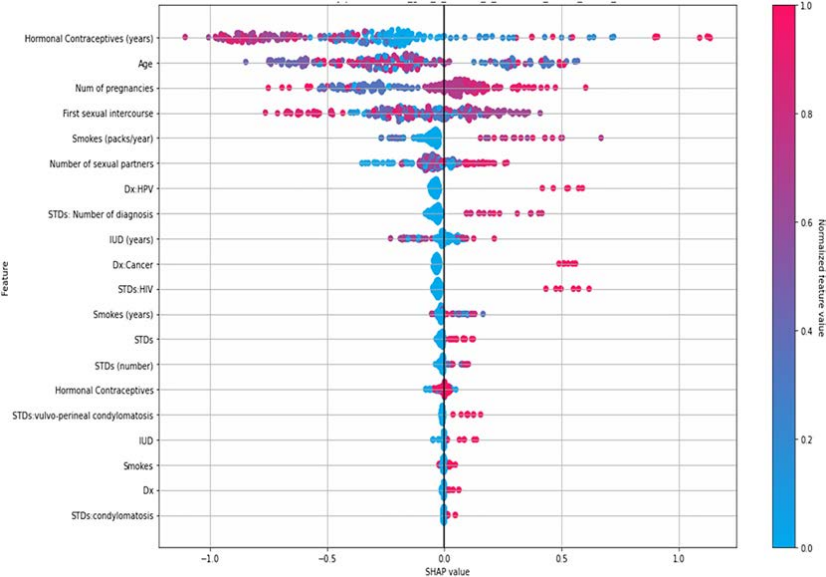
SHAP explainability values of each feature.

Fig. 9, depicts the SHAP summary plot, which consists of a comprehensive visualization of feature importance in a DT model designed to classify cervical cancer. The *x*-axis represents the SHAP values, which quantify the impact of each feature on the model's output, with positive values indicating a higher likelihood of cervical cancer and negative values indicating a lower likelihood, whereas the *y*-axis lists the features analyzed. In this figure, all features rank indicated by color code from low feature value (blue) to high feature value (red). ‘Hormonal Contraceptives (years),’ which exhibits the highest positive impact to successfully classify cervical cancer and its effect on the model's output, with SHAP values extending up to 1. In addition, ‘Age’ also shows a significant positive impact, particularly at higher values just over 0.5. ‘Number of pregnancies’, ‘First sexual intercourse’, and ‘Smoke(packs/year)’ have varied impacts, suggesting complex relationships with the target variable. Other features like ‘IUD (years)’, ‘Smokes (years)’, and ‘STDs’ show moderate effects.

## Conclusion

Despite substantial medical advancements, clinicians are finding it more difficult to eradicate the prevalence of cervical cancer mortality rates. Hence, this study presents a machine learning-based system that can detect the early stage of cervical cancer patients depending on the risk factors of cervical cancer. In this research, 858 individuals with 36 distinct attributes were used, which were collected from the UCI machine learning repository. During the data preparation stage, we eliminated missing values by calculating mean, and applied SMOTE and ADASYN techniques to address the issue of data imbalance. Subsequently, Chi-square and LASSO feature selection were used to evaluate the most significant features, which are mostly correlated to identify the particular disease. In addition, six supervised machine learning models have been performed in this research, where the DT algorithm outperformed with 97.60% accuracy, 98.73% sensitivity and 80% specificity for Chi-square feature selection. By contrast, DT gained 97% accuracy, 99.35% sensitivity and 69.23% specificity for the imbalanced dataset. Furthermore, the explainable AI technique (SHAP) was used to extend and check the validity of the model outcome precisely. In the future, we want to extend this study by including a larger real-time dataset and reducing the computation cost. In addition, we will choose some different techniques of XAI to make this ML model more robust.

## Declaration of competing interest

The authors declare that they have no known competing financial interests or personal relationships that could have appeared to influence the work reported in this article.
